# 2,7-Dimethoxy­-1-(4-nitro­benzo­yl)-naphthalene

**DOI:** 10.1107/S1600536810005398

**Published:** 2010-02-13

**Authors:** Shoji Watanabe, Kosuke Nakaema, Takahiro Nishijima, Akiko Okamoto, Noriyuki Yonezawa

**Affiliations:** a Department of Organic and Polymer Materials Chemistry, Tokyo University of Agriculture and Technology, Koganei, Tokyo 184-8588, Japan

## Abstract

In the title compound, C_19_H_15_NO_5_, the dihedral angle between the naphthalene ring system and the benzene ring is 61.97 (5)°. The dihedral between the naphthalene ring system and the bridging carbonyl C—C(=O)—C plane is 54.68 (6)°, far larger than that [12.54 (7)°] between the phenyl group and the bridging carbonyl group. The nitro group and the phenyl ring are almost coplanar [O—N—C—C torsion angle = 2.94 (19)°]. In the crystal, mol­ecules are linked by C—H⋯π inter­actions and the phenyl rings are involved in a centrosymmetric π–π inter­action with a perpendicular distance of 3.523 Å and a lateral offset of 1.497 Å. In addition, weak inter­molecular C—H⋯O hydrogen bonds are formed between an H atom of one meth­oxy group and a nearby carbonyl O atom.

## Related literature

For general background to the regioselective formation of *peri*-aroylnaphthalene compounds, see: Okamoto & Yonezawa (2009 ▶). For related structures, see: Mitsui *et al.* (2008 ▶, 2009 ▶); Nakaema *et al.* (2007 ▶, 2008 ▶); Watanabe *et al.* (2010*a* ▶,*b* ▶).
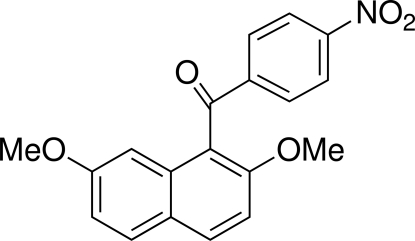


## Experimental

### 

#### Crystal data

C_19_H_15_NO_5_
*M* *_r_* = 337.32
Monoclinic, 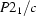

*a* = 8.6877 (6) Å
*b* = 28.870 (2) Å
*c* = 6.4635 (5) Å
β = 90.839 (5)°
*V* = 1621.0 (2) Å^3^
*Z* = 4
Cu *K*α radiation
μ = 0.84 mm^−1^
*T* = 296 K
0.60 × 0.60 × 0.20 mm

#### Data collection

Rigaku R-AXIS RAPID diffractometer
Absorption correction: numerical (*NUMABS*; Higashi, 1999 ▶) *T*_min_ = 0.632, *T*_max_ = 0.850
29623 measured reflections
2954 independent reflections
2713 reflections with *I* > 2σ(*I*)
*R*_int_ = 0.033

#### Refinement

*R*[*F* ^2^ > 2σ(*F* ^2^)] = 0.036
*wR*(*F* ^2^) = 0.102
*S* = 1.05
2954 reflections
229 parameters
H-atom parameters constrained
Δρ_max_ = 0.17 e Å^−3^
Δρ_min_ = −0.19 e Å^−3^

### 

Data collection: *PROCESS-AUTO* (Rigaku, 1998 ▶); cell refinement: *PROCESS-AUTO*; data reduction: *CrystalStructure* (Rigaku/MSC, 2004 ▶); program(s) used to solve structure: *SIR2004* (Burla *et al.*, 2005 ▶); program(s) used to refine structure: *SHELXL97* (Sheldrick, 2008 ▶); molecular graphics: *ORTEPIII* (Burnett & Johnson, 1996 ▶); software used to prepare material for publication: *SHELXL97*.

## Supplementary Material

Crystal structure: contains datablocks global, I. DOI: 10.1107/S1600536810005398/fl2290sup1.cif

Structure factors: contains datablocks I. DOI: 10.1107/S1600536810005398/fl2290Isup2.hkl

Additional supplementary materials:  crystallographic information; 3D view; checkCIF report

## Figures and Tables

**Table 1:** Hydrogen-bond geometry (Å, °) *Cg*1 is the centroid of the naphthalene ring system C1–C5,C10.

*D*—H⋯*A*	*D*—H	H⋯*A*	*D*⋯*A*	*D*—H⋯*A*
C3—H3⋯*Cg*1^i^	0.93	2.81	3.5789 (15)	141
C19—H19*B*⋯*Cg*1^ii^	0.96	2.91	3.7605 (19)	148
C18—H18*C*⋯O1^iii^	0.96	2.49	3.281 (2)	140

Symmetry codes: (i) 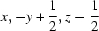; (ii) 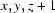; (iii) 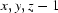.

**Fig. 1.**
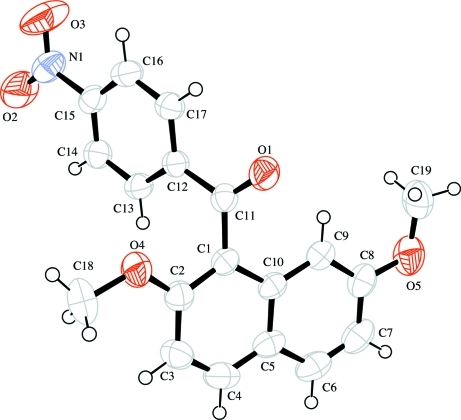
Molecular structure of (I), with the atom-labeling scheme and displacement ellipsoids drawn at the 50% probability level.

**Fig. 2.**
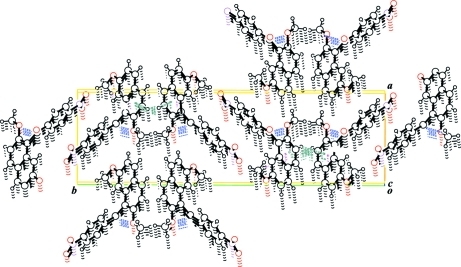
The alignment of the molecules in the crystal structure, viewed along c axis.

**Fig. 3.**
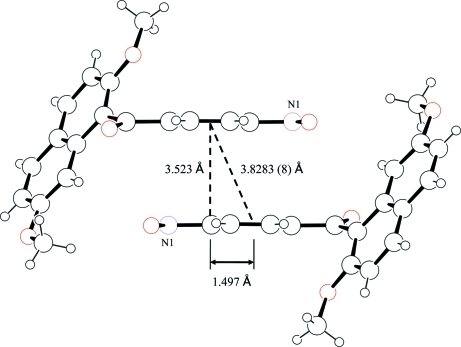
Side-on view of the π–π interaction.

**Fig. 4.**
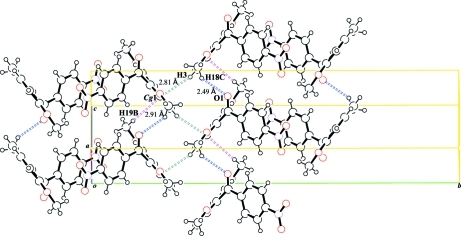
Two types of C—H···π interactions (Green Copper dotted lines, Violet Red dotted lines) and weak C—H···O interactions (Blue dotted lines).

## supplementary crystallographic information

### Comment

In the course of our study on electrophilic aromatic aroylation of 2,7-dimethoxynaphthalene, *peri*-aroylnaphthalene compounds have proven to be formed regioselectively with the aid of suitable acidic mediators (Okamoto & Yonezawa, 2009). The aroyl groups at the 1,8-positions of the naphthalene rings in these compounds are twisted almost perpendicularly but the benzene ring moieties of the aroyl groups tilt slightly toward the exo sides of the naphthalene rings. Recently, we reported the structures of 1,8-diaroyl-2,7-dimethoxynaphthalenes, i. e., 1,8-bis(4-chlorobenzoyl)-2,7-dimethoxynaphthalene (Nakaema *et al.*, 2007), 1,8-dibenzoyl-2,7-dimethoxynaphthalene (Nakaema *et al.*, 2008), (2,7-dimethoxynaphthalene-1,8-diyl)bis(4-fluorobenzoyl)dimethanone (Watanabe *et al.*, 2010a) and bis(4-bromobenzoyl)(2,7-dimethoxynaphthalene-1,8-diyl)dimethanone (Watanabe *et al.*, 2010b). Furthermore, the crystal structures of 1-aroyl-2,7-dimethoxynaphthalenes, i. e., 1-(4-chlorobenzoyl)-2,7-dimethoxynaphthalene (Mitsui *et al.*, 2008) and (4-chlorobenzoyl)(2-ethoxy-7-methoxynaphthalen-1-yl)methanone (Mitsui *et al*. 2009), also exhibit essentially the same non-coplanar structure as the 1,8-diaroylated naphthalenes. As a part of our ongoing studies on the formation and the structure of the aroylated naphthalene derivatives, the synthesis and crystal structure of (I), a 1-monoaroylnaphthalene bearing nitro group, is discussed in this report. (I) was prepared by electrophilic aromatic aroylation reaction of 2,7-dimethoxynaphthalene with 4-nitrobenzoyl chloride.

The molecular structure of (I) is displayed in Fig. 1. The interplanar angle between the benzene ring (C12—C17) and the naphthalene ring (C1—C10) is 61.97 (5)°. The dihedral angle between the carbonyl and the naphthalene is 54.68 (6)° [C10—C1—C11—O1 torsion angle = -53.67 (17)°]. On the other hand, the dihedral angle between the carbonyl group and the phenyl ring is 12.54 (7)° [O1—C11—C12—C17 torsion angle = -13.29 (17)°]. The nitro group and the phenyl group are almost coplanar [O3—N1—C15—C14 torsion angle = 2.94 (19)°].

The molecular packing of (I) is mainly stabilized by van der Waals interactions. The molecules are aligned consecutively in stacks along the *c* axis (Fig. 2). Adjacent 4-nitrophenyl groups related by crystallographic inversion centers are exactly antiparallel and the perpendicular distance between the mean planes is 3.523 Å (Fig. 3). The centroid-centroid distance between the two antiparallel phenyl rings is 3.8283 (8) Å and the lateral offset is 1.497 Å, indicating the presence of a π–π interaction.

Moreover, molecules are linked by two types of C—H···π interactions. The naphthalene ring acts as a hydrogen-bond donor and the π system of the naphthalene ring [C1—C10 ring (with centroid *Cg*1)] of an adjacent molecule acts as an accepter (C3—H3···π^i^) (Fig. 4). The methyl group acts as a hydrogen-bond donor and the π system of the naphthalene ring [C1—C10 ring (with centroid *Cg*1)] of an adjacent molecule acts as an accepter (C19—H19C···π^ii^) .

The crystal packing is additionally stabilized by intermolecular weak C—H···O hydrogen bonding between the carbonyl oxygen and a hydrogen atom of a nearby methyl group (C18—H18B···O1^iii^; Fig. 4 and Table 1).

### Experimental

The title compound was prepared by treatment of a mixture of 2,7-dimethoxynaphthalene (1.0 mmol) and 4-nitrobenzoic acid (2.2 mmol) with phosphorus pentoxide–methanesulfonic acid mixture (P_2_O_5_–MsOH [1/10 w/w]; 4.4 ml) at 40°C for 0.5 hours followed by a typical work-up procedure (50% yield; Okamoto & Yonezawa, 2009). Yellow platelet single crystals suitable for X-ray diffraction were obtained by recrystallization from chloroform.

Spectroscopic Data: ^1^H NMR (300 MHz, CDCl_3_) δ 3.76 (3H, s), 3.76 (3H, s), 6.87 (1H, d, *J* = 2.3 Hz), 7.05 (1H, dd, *J* = 9.0, 2.3 Hz), 7.16 (1H, d, *J* = 9.0 Hz), 7.75 (1H, d, *J* = 8.7 Hz), 7.93 (1H, d, *J* = 9.0 Hz), 7.98 (2H, d, *J* = 9.0 Hz), 8.27 (2H, d, *J* = 8.7 Hz); ^13^C NMR (75 MHz, CDCl_3_) δ 21.8, 22.7, 68.3, 76.4, 82.5, 83.9, 90.3, 91.0, 95.7, 96.5, 96.7, 98.8, 99.6, 109.6, 122.3, 126.0, 162.9; IR (KBr): 1357, 1594, 1267; Anal. Calcd for C_19_H_15_NO_5_: C 67.65, H 4.48, N 4.15. Found: C 67.52, H 4.51, N 4.06.

### Refinement

All the H atoms were found in difference maps and were subsequently refined as riding atoms, with C—H = 0.93 (aromatic) and 0.96 (methyl) Å, and U_iso_(H) = 1.2U_eq_(C).

### Crystal data

**Table:**

C_19_H_15_NO_5_	*F*(000) = 704
*M**_r_* = 337.32	*D*_x_ = 1.382 Mg m^−^^3^
Monoclinic, *P*2_1_/*c*	Melting point: 440 K
Hall symbol: -P 2ybc	Cu *K*α radiation, λ = 1.54187 Å
*a* = 8.6877 (6) Å	Cell parameters from 28804 reflections
*b* = 28.870 (2) Å	θ = 3.1–68.2°
*c* = 6.4635 (5) Å	µ = 0.84 mm^−^^1^
β = 90.839 (5)°	*T* = 296 K
*V* = 1621.0 (2) Å^3^	Platelet, yellow
*Z* = 4	0.60 × 0.60 × 0.20 mm

### Data collection

**Table:**

Rigaku R-AXIS RAPID diffractometer	2954 independent reflections
Radiation source: rotating anode	2713 reflections with *I* > 2σ(*I*)
graphite	*R*_int_ = 0.033
Detector resolution: 10.00 pixels mm^-1^	θ_max_ = 68.2°, θ_min_ = 3.1°
ω scans	*h* = −10→10
Absorption correction: numerical (*NUMABS*; Higashi, 1999)	*k* = −34→34
*T*_min_ = 0.632, *T*_max_ = 0.850	*l* = −7→7
29623 measured reflections	

### Refinement

**Table:**

Refinement on *F*^2^	Secondary atom site location: difference Fourier map
Least-squares matrix: full	Hydrogen site location: difference Fourier map
*R*[*F*^2^ > 2σ(*F*^2^)] = 0.036	H-atom parameters constrained
*wR*(*F*^2^) = 0.102	*w* = 1/[σ^2^(*F*_o_^2^) + (0.0491*P*)^2^ + 0.3261*P*] where *P* = (*F*_o_^2^ + 2*F*_c_^2^)/3
*S* = 1.05	(Δ/σ)_max_ < 0.001
2954 reflections	Δρ_max_ = 0.17 e Å^−^^3^
229 parameters	Δρ_min_ = −0.18 e Å^−^^3^
0 restraints	Extinction correction: *SHELXL97* (Sheldrick, 2008), Fc^*^=kFc[1+0.001xFc^2^λ^3^/sin(2θ)]^-1/4^
Primary atom site location: structure-invariant direct methods	Extinction coefficient: 0.0064 (5)

### Special details

**Table:**

Geometry. All esds (except the esd in the dihedral angle between two l.s. planes) are estimated using the full covariance matrix. The cell esds are taken into account individually in the estimation of esds in distances, angles and torsion angles; correlations between esds in cell parameters are only used when they are defined by crystal symmetry. An approximate (isotropic) treatment of cell esds is used for estimating esds involving l.s. planes.
Refinement. Refinement of *F*^2^ against ALL reflections. The weighted *R*-factor wR and goodness of fit *S* are based on *F*^2^, conventional *R*-factors *R* are based on *F*, with *F* set to zero for negative *F*^2^. The threshold expression of *F*^2^ > σ(*F*^2^) is used only for calculating *R*-factors(gt) etc. and is not relevant to the choice of reflections for refinement. *R*-factors based on *F*^2^ are statistically about twice as large as those based on *F*, and *R*- factors based on ALL data will be even larger.

### Fractional atomic coordinates and isotropic or equivalent isotropic displacement parameters (Å^2^)

**Table:**

	*x*	*y*	*z*	*U*_iso_*/*U*_eq_	
O1	0.46668 (12)	0.12949 (4)	0.41178 (15)	0.0654 (3)	
O2	0.72324 (18)	−0.01707 (5)	−0.4151 (2)	0.0998 (5)	
O3	0.86695 (18)	−0.03032 (5)	−0.1503 (3)	0.1103 (5)	
O4	0.52962 (12)	0.18647 (4)	−0.08228 (16)	0.0628 (3)	
O5	−0.08753 (12)	0.11905 (5)	0.59355 (19)	0.0765 (3)	
N1	0.76264 (15)	−0.00977 (4)	−0.2367 (2)	0.0655 (3)	
C1	0.32998 (14)	0.15939 (4)	0.12073 (19)	0.0443 (3)	
C2	0.37575 (16)	0.18672 (4)	−0.0431 (2)	0.0507 (3)	
C3	0.26878 (19)	0.21395 (5)	−0.1541 (2)	0.0639 (4)	
H3	0.3001	0.2317	−0.2659	0.077*	
C4	0.1186 (2)	0.21401 (5)	−0.0961 (3)	0.0652 (4)	
H4	0.0477	0.2313	−0.1726	0.078*	
C5	0.06755 (16)	0.18894 (4)	0.0748 (2)	0.0532 (3)	
C6	−0.08761 (17)	0.19031 (5)	0.1403 (3)	0.0640 (4)	
H6	−0.1591	0.2076	0.0648	0.077*	
C7	−0.13369 (16)	0.16718 (6)	0.3093 (3)	0.0650 (4)	
H7	−0.2357	0.1689	0.3501	0.078*	
C8	−0.02757 (16)	0.14036 (5)	0.4242 (2)	0.0569 (4)	
C9	0.12269 (15)	0.13696 (4)	0.3656 (2)	0.0492 (3)	
H9	0.1909	0.1185	0.4413	0.059*	
C10	0.17471 (14)	0.16139 (4)	0.19014 (19)	0.0445 (3)	
C11	0.44394 (14)	0.12808 (4)	0.22566 (19)	0.0447 (3)	
C12	0.52624 (13)	0.09236 (4)	0.09933 (18)	0.0418 (3)	
C13	0.48124 (15)	0.08167 (4)	−0.1027 (2)	0.0481 (3)	
H13	0.3990	0.0974	−0.1642	0.058*	
C14	0.55780 (16)	0.04787 (5)	−0.2128 (2)	0.0513 (3)	
H14	0.5281	0.0405	−0.3477	0.062*	
C15	0.67883 (15)	0.02546 (4)	−0.1175 (2)	0.0488 (3)	
C16	0.72680 (15)	0.03499 (5)	0.0825 (2)	0.0529 (3)	
H16	0.8093	0.0192	0.1426	0.063*	
C17	0.64890 (14)	0.06867 (4)	0.1911 (2)	0.0481 (3)	
H17	0.6787	0.0755	0.3264	0.058*	
C18	0.5831 (2)	0.20964 (7)	−0.2619 (2)	0.0781 (5)	
H18A	0.6892	0.2019	−0.2832	0.094*	
H18B	0.5734	0.2425	−0.2436	0.094*	
H18C	0.5228	0.2001	−0.3801	0.094*	
C19	0.0143 (2)	0.09324 (6)	0.7227 (3)	0.0767 (5)	
H19A	−0.0384	0.0838	0.8452	0.092*	
H19B	0.1012	0.1121	0.7607	0.092*	
H19C	0.0490	0.0663	0.6497	0.092*	

### Atomic displacement parameters (Å^2^)

**Table:**

	*U*^11^	*U*^22^	*U*^33^	*U*^12^	*U*^13^	*U*^23^
O1	0.0595 (6)	0.0914 (8)	0.0453 (5)	0.0172 (5)	−0.0034 (4)	−0.0093 (5)
O2	0.1225 (12)	0.1010 (10)	0.0759 (9)	0.0427 (8)	0.0043 (8)	−0.0265 (7)
O3	0.0969 (10)	0.1002 (10)	0.1330 (13)	0.0545 (8)	−0.0240 (9)	−0.0403 (9)
O4	0.0602 (6)	0.0626 (6)	0.0659 (6)	−0.0045 (5)	0.0160 (5)	0.0089 (5)
O5	0.0524 (6)	0.0989 (9)	0.0787 (8)	−0.0120 (6)	0.0186 (5)	0.0010 (6)
N1	0.0615 (8)	0.0523 (7)	0.0828 (9)	0.0089 (6)	0.0063 (7)	−0.0100 (6)
C1	0.0460 (7)	0.0403 (6)	0.0467 (7)	0.0025 (5)	0.0017 (5)	−0.0048 (5)
C2	0.0564 (8)	0.0431 (6)	0.0527 (7)	−0.0005 (5)	0.0056 (6)	−0.0032 (5)
C3	0.0813 (11)	0.0499 (8)	0.0606 (9)	0.0052 (7)	0.0037 (7)	0.0109 (6)
C4	0.0716 (10)	0.0545 (8)	0.0690 (9)	0.0141 (7)	−0.0105 (8)	0.0069 (7)
C5	0.0526 (8)	0.0455 (7)	0.0612 (8)	0.0076 (5)	−0.0068 (6)	−0.0089 (6)
C6	0.0481 (8)	0.0628 (9)	0.0806 (10)	0.0124 (6)	−0.0127 (7)	−0.0139 (8)
C7	0.0385 (7)	0.0716 (9)	0.0850 (11)	0.0003 (6)	0.0025 (7)	−0.0194 (8)
C8	0.0462 (7)	0.0607 (8)	0.0639 (9)	−0.0073 (6)	0.0069 (6)	−0.0128 (7)
C9	0.0440 (7)	0.0497 (7)	0.0539 (7)	0.0009 (5)	0.0022 (5)	−0.0052 (6)
C10	0.0446 (7)	0.0390 (6)	0.0498 (7)	0.0025 (5)	−0.0012 (5)	−0.0092 (5)
C11	0.0393 (6)	0.0501 (7)	0.0446 (7)	−0.0017 (5)	0.0020 (5)	−0.0009 (5)
C12	0.0385 (6)	0.0413 (6)	0.0455 (6)	−0.0021 (5)	0.0026 (5)	0.0037 (5)
C13	0.0451 (7)	0.0506 (7)	0.0486 (7)	0.0068 (5)	−0.0027 (5)	0.0007 (5)
C14	0.0549 (7)	0.0511 (7)	0.0478 (7)	0.0018 (6)	0.0011 (6)	−0.0025 (5)
C15	0.0464 (7)	0.0401 (6)	0.0602 (8)	0.0003 (5)	0.0092 (6)	−0.0004 (5)
C16	0.0456 (7)	0.0482 (7)	0.0647 (8)	0.0066 (5)	−0.0025 (6)	0.0067 (6)
C17	0.0467 (7)	0.0495 (7)	0.0480 (7)	0.0002 (5)	−0.0024 (5)	0.0047 (5)
C18	0.0854 (12)	0.0950 (12)	0.0545 (9)	−0.0255 (10)	0.0178 (8)	−0.0001 (8)
C19	0.0776 (11)	0.0812 (11)	0.0720 (11)	−0.0124 (9)	0.0217 (9)	0.0066 (9)

### Geometric parameters (Å, °)

**Table:**

O1—C11	1.2169 (15)	C7—H7	0.9300
O2—N1	1.2163 (18)	C8—C9	1.3681 (19)
O3—N1	1.2126 (18)	C9—C10	1.4151 (18)
O4—C2	1.3641 (17)	C9—H9	0.9300
O4—C18	1.4239 (18)	C11—C12	1.5027 (17)
O5—C8	1.3656 (18)	C12—C17	1.3917 (17)
O5—C19	1.419 (2)	C12—C13	1.3923 (18)
N1—C15	1.4748 (17)	C13—C14	1.3837 (18)
C1—C2	1.3837 (18)	C13—H13	0.9300
C1—C10	1.4290 (17)	C14—C15	1.3730 (19)
C1—C11	1.4960 (17)	C14—H14	0.9300
C2—C3	1.406 (2)	C15—C16	1.380 (2)
C3—C4	1.363 (2)	C16—C17	1.3820 (19)
C3—H3	0.9300	C16—H16	0.9300
C4—C5	1.398 (2)	C17—H17	0.9300
C4—H4	0.9300	C18—H18A	0.9600
C5—C6	1.420 (2)	C18—H18B	0.9600
C5—C10	1.4265 (18)	C18—H18C	0.9600
C6—C7	1.346 (2)	C19—H19A	0.9600
C6—H6	0.9300	C19—H19B	0.9600
C7—C8	1.408 (2)	C19—H19C	0.9600
			
C2—O4—C18	118.76 (13)	C5—C10—C1	118.05 (12)
C8—O5—C19	117.80 (12)	O1—C11—C1	121.65 (11)
O3—N1—O2	123.34 (14)	O1—C11—C12	119.24 (11)
O3—N1—C15	117.93 (14)	C1—C11—C12	119.03 (10)
O2—N1—C15	118.72 (13)	C17—C12—C13	119.56 (11)
C2—C1—C10	120.03 (12)	C17—C12—C11	118.24 (11)
C2—C1—C11	119.73 (11)	C13—C12—C11	122.17 (11)
C10—C1—C11	120.24 (11)	C14—C13—C12	120.55 (12)
O4—C2—C1	115.61 (12)	C14—C13—H13	119.7
O4—C2—C3	123.40 (12)	C12—C13—H13	119.7
C1—C2—C3	120.94 (13)	C15—C14—C13	118.17 (12)
C4—C3—C2	119.23 (13)	C15—C14—H14	120.9
C4—C3—H3	120.4	C13—C14—H14	120.9
C2—C3—H3	120.4	C14—C15—C16	123.05 (12)
C3—C4—C5	122.22 (14)	C14—C15—N1	118.11 (12)
C3—C4—H4	118.9	C16—C15—N1	118.83 (12)
C5—C4—H4	118.9	C15—C16—C17	118.19 (12)
C4—C5—C6	122.36 (14)	C15—C16—H16	120.9
C4—C5—C10	119.29 (13)	C17—C16—H16	120.9
C6—C5—C10	118.34 (14)	C16—C17—C12	120.47 (12)
C7—C6—C5	121.57 (14)	C16—C17—H17	119.8
C7—C6—H6	119.2	C12—C17—H17	119.8
C5—C6—H6	119.2	O4—C18—H18A	109.5
C6—C7—C8	120.05 (13)	O4—C18—H18B	109.5
C6—C7—H7	120.0	H18A—C18—H18B	109.5
C8—C7—H7	120.0	O4—C18—H18C	109.5
O5—C8—C9	124.50 (14)	H18A—C18—H18C	109.5
O5—C8—C7	114.60 (13)	H18B—C18—H18C	109.5
C9—C8—C7	120.90 (14)	O5—C19—H19A	109.5
C8—C9—C10	120.22 (13)	O5—C19—H19B	109.5
C8—C9—H9	119.9	H19A—C19—H19B	109.5
C10—C9—H9	119.9	O5—C19—H19C	109.5
C9—C10—C5	118.89 (12)	H19A—C19—H19C	109.5
C9—C10—C1	123.06 (11)	H19B—C19—H19C	109.5
			
C18—O4—C2—C1	173.20 (13)	C2—C1—C10—C9	−175.40 (12)
C18—O4—C2—C3	−9.2 (2)	C11—C1—C10—C9	4.15 (18)
C10—C1—C2—O4	172.62 (11)	C2—C1—C10—C5	5.40 (17)
C11—C1—C2—O4	−6.93 (17)	C11—C1—C10—C5	−175.06 (11)
C10—C1—C2—C3	−5.08 (19)	C2—C1—C11—O1	125.86 (14)
C11—C1—C2—C3	175.38 (12)	C10—C1—C11—O1	−53.69 (17)
O4—C2—C3—C4	−176.15 (14)	C2—C1—C11—C12	−57.33 (16)
C1—C2—C3—C4	1.4 (2)	C10—C1—C11—C12	123.12 (12)
C2—C3—C4—C5	2.0 (2)	O1—C11—C12—C17	−13.28 (17)
C3—C4—C5—C6	177.57 (14)	C1—C11—C12—C17	169.84 (11)
C3—C4—C5—C10	−1.5 (2)	O1—C11—C12—C13	165.11 (12)
C4—C5—C6—C7	−177.73 (14)	C1—C11—C12—C13	−11.77 (17)
C10—C5—C6—C7	1.4 (2)	C17—C12—C13—C14	−0.26 (19)
C5—C6—C7—C8	−0.8 (2)	C11—C12—C13—C14	−178.63 (11)
C19—O5—C8—C9	2.9 (2)	C12—C13—C14—C15	−0.14 (19)
C19—O5—C8—C7	−176.97 (14)	C13—C14—C15—C16	0.3 (2)
C6—C7—C8—O5	179.19 (13)	C13—C14—C15—N1	−178.58 (12)
C6—C7—C8—C9	−0.7 (2)	O3—N1—C15—C14	−178.16 (15)
O5—C8—C9—C10	−178.35 (12)	O2—N1—C15—C14	1.2 (2)
C7—C8—C9—C10	1.5 (2)	O3—N1—C15—C16	2.9 (2)
C8—C9—C10—C5	−0.86 (18)	O2—N1—C15—C16	−177.70 (14)
C8—C9—C10—C1	179.93 (12)	C14—C15—C16—C17	0.0 (2)
C4—C5—C10—C9	178.59 (12)	N1—C15—C16—C17	178.86 (11)
C6—C5—C10—C9	−0.53 (18)	C15—C16—C17—C12	−0.44 (19)
C4—C5—C10—C1	−2.16 (18)	C13—C12—C17—C16	0.55 (18)
C6—C5—C10—C1	178.71 (11)	C11—C12—C17—C16	178.99 (11)

### Hydrogen-bond geometry (Å, °)

**Table:**

Cg1 is the centroid of the naphthalene ring system C1–C10.

**Table:**

*D*—H···*A*	*D*—H	H···*A*	*D*···*A*	*D*—H···*A*
C3—H3···Cg1^i^	0.93	2.81	3.5789 (15)	141
C19—H19B···Cg1^ii^	0.96	2.91	3.7605 (19)	148
C18—H18C···O1^iii^	0.96	2.49	3.281 (2)	140

Symmetry codes: (i) *x*, −*y*+1/2, *z*−1/2; (ii) *x*, *y*, *z*+1; (iii) *x*, *y*, *z*−1.
